# In vitro analysis of dimensional stability of fast-setting irreversible hydrocolloids by digital scanning

**DOI:** 10.1590/1807-3107bor-2026.vol40.087

**Published:** 2026-11-09

**Authors:** Mariana Coutinho SANCAS, Ido Luiz de Azevedo FEITEN, Ana Carolina SANTAREM, Thalita de Paris MATOS, Michael Willian FAVORETO, Larissa CAVALCANTE, Luis Felipe SCHNEIDER

**Affiliations:** (a) Universidade Federal do Rio de Janeiro – UFRJ, School of Dentistry, Department of Pediatric Dentistry and Orthodontics, Rio de Janeiro, RJ, Brazil.; (b) Universidade Federal Fluminense – UFF, School of Dentistry, Department of Dental Technique, Niterói, RJ, Brazil.; (c) DFL Indústria e Comércio SA – DFL, Department of Clinical Research, Rio de Janeiro, RJ, Brazil.; (d) Universidade Federal do Paraná - UFPR, School of Dentistry, Department of Restorative Dentistry, Curitiba, PR, Brazil.; (e) Universidade de São Paulo – USP, School of Dentistry, Department of Restorative Dentistry, São Paulo, SP, Brazil.

**Keywords:** Dental Impression Materials, Alginates

## Abstract

This study aimed to evaluate the *in vitro* dimensional stability of Moldeer Fast compared with two commercially available extended-pour, fast-setting irreversible hydrocolloids using direct digital scanning of dental impressions. This experimental *in vitro* study was conducted using standardized impression techniques on a typodont. Impressions were obtained in random order by a single calibrated operator. Three irreversible hydrocolloids were evaluated: ColorChange (Cavex Holland BV), Hydrogum 5 (Zhermack Spa), and Moldeer Fast (DFL). Ten impressions were obtained per group according to the manufacturers’ instructions and scanned using an intraoral scanner at predetermined intervals (0 h, 24 h, 48 h, 72 h, 96 h, and 120 h). Impressions were stored under controlled temperature and humidity conditions. Four linear measurements were performed on color-masked images in a blind manner to assess dimensional changes over time: a) intercanine distance, b) intermolar distance, c) right canine-to-molar distance, and d) left canine-to-molar distance. Data were analyzed using repeated-measures ANOVA (α = 0.05). All materials exhibited minor dimensional changes, consisting predominantly of slight contraction. However, no statistically significant differences were observed over time (*p* > 0.05). Moldeer Fast therefore exhibited dimensional stability comparable to that of ColorChange and Hydrogum 5. All materials maintained clinically acceptable dimensional stability for up to five days, supporting their suitability for delayed digital scanning of irreversible hydrocolloid impressions.

## Introduction

Despite significant advances in digital workflows in clinical dentistry, many practitioners still prefer conventional analog impressions or consider it premature to invest in intraoral scanning technology.^1^ Among the available impression materials, irreversible hydrocolloids (commonly known as alginates) remain among the most widely used due to their ease of manipulation, lower cost than that of elastomeric materials, and lack of a need for specialized equipment or extensive training.^2,3^

However, irreversible hydrocolloid impressions are susceptible to dimensional changes resulting from water loss or uptake through syneresis and imbibition, respectively.^4,5^ Significant fluctuations in water content during handling or storage may cause dimensional distortion and compromise accuracy.^6^ Traditionally, the standard recommendation has been to pour the cast immediately after the impression is removed from the oral cavity.^2,7^ Nevertheless, immediate cast fabrication may be limited by logistical constraints or clinical workflow demands.

To overcome this limitation while preserving dimensional stability, manufacturers have developed extended-pour irreversible hydrocolloids—formulations with greater resistance to syneresis and imbibition that permit delayed pouring without clinically significant distortion^6^. Some of these materials incorporate a chromatic phase indicator to assist clinicians in identifying the setting stage and can also be digitized, thereby facilitating integration into digital workflows.^3,8^

The incorporation of three-dimensional (3D) digital models represents a substantial advance in dentistry by overcoming several limitations of traditional gypsum casts, including their space-intensive storage requirements and susceptibility to damage over time.^9^ Digital models improve data retrieval, facilitate interdisciplinary communication, and provide accurate linear measurements for research, treatment planning, and patient monitoring.^3,9,10^ The lack of standardized dimensional stability analyses under digital scanning conditions therefore represents an important clinical and methodological gap that remains to be addressed.

However, barriers to the widespread adoption of intraoral scanners remain. These include the high cost of the equipment, the learning curve associated with both software and hardware, and potentially longer acquisition times compared with conventional irreversible hydrocolloid impressions, particularly when the latter are performed by experienced clinicians.^9^ Moreover, intraoral scanners may pose logistical challenges in multi-operatory clinical settings. In such situations, scan-compatible irreversible hydrocolloids with prolonged dimensional stability provide a valuable alternative for preliminary data collection, study model fabrication, and digital data acquisition.

Although several extended-pour irreversible hydrocolloids are commercially available, concerns regarding their dimensional stability persist. Among the most extensively investigated materials are ColorChange (Cavex Holland BV, Haarlem, The Netherlands) and Hydrogum 5 (Zhermack SpA, Badia Polesine, Italy), both of which have demonstrated satisfactory clinical performance in multiple studies^2,5^. However, findings regarding their dimensional accuracy over time remain inconsistent, likely due to heterogeneity in experimental protocols.

Against this background, Moldeer Fast (DFL, Rio de Janeiro, Brazil), a new irreversible hydrocolloid, has recently been introduced. According to the manufacturer, its formulation was designed to combine the desirable characteristics of an alginate impression material, including rapid setting, extended-pour capability, a chromatic phase indicator, and compatibility with digital scanning. The manufacturer also states that the material provides dimensional stability comparable to that of high-precision irreversible hydrocolloids. However, no independent studies have evaluated its performance, particularly under digital scanning conditions.

Given the limited number of studies evaluating the dimensional stability of extended-pour irreversible hydrocolloids using digital scanning methods, together with the recent introduction of Moldeer Fast, this study aimed to assess its in vitro dimensional stability compared with two well-established extended-pour, fast-setting irreversible hydrocolloids over a 120-hour period. The null hypothesis was that no statistically significant differences in dimensional stability would be observed among the tested materials over time.

## Methods

### Study design and experimental groups

This in vitro experimental study evaluated the dimensional stability of three extended-pour fast-setting irreversible hydrocolloids: ColorChange (Cavex Holland BV, Haarlem, The Netherlands), Hydrogum 5 (Zhermack SpA, Badia Polesine, Italy), and Moldeer Fast (DFL, Rio de Janeiro, Brazil). The tested materials and their main characteristics are summarized in Table 1.

**Table 1:** Characteristics of the tested irreversible hydrocolloids.

Product	Manufacturer	Setting time	Chromatic	Stability (days)	Composition	Flavor	Batch
ColorChange	Cavex Holland BV, Haarlem, The Netherlands	2 minutes 30 seconds	Yes	9	Alginate, calcium sulfate, fillers, retarder, stabilizers, flavor, color indicators	Mint	240411
Hydrogum 5	Zhermack SpA, Badia Polesine, Italy	1 minute 50 seconds	No	5	Diatomaceous earth, calcium sulfate, potassium alginate, sodium pyrophosphate, potassium fluorotitanate, magnesium hydroxide, petroleum jelly, pigments and flavoring agents	Mangosteen	C302070
Moldeer Fast	DFL, Rio de Janeiro, Brazil	2 minutes	Yes	5	Sodium alginate, zinc oxide, calcium sulfate dihydrate, diatomaceous earth, aluminum hydroxide, potassium hexafluorotitanate	Mint	24086

### Preparation of specimens

All specimens were prepared manually by the same trained operator (MCS) according to the manufacturers’ instructions. To obtain 30 impressions (n = 10 per group), the order of specimen preparation was randomized using a coded allocation list generated in advance by a second researcher (IF). Before dispensing, each bag was gently shaken to homogenize the irreversible hydrocolloid powder. Using the manufacturer’s measuring scoop, the powder was collected without compression and leveled with a flat spatula to remove excess material. All bags remained sealed when not in use.

A maxillary typodont (N51, P-OCLUSAL Produtos Odontológicos, São Paulo, Brazil) was selected to simulate clinical conditions. The protocol was standardized by using two scoops of irreversible hydrocolloid powder and two measures of distilled water for each impression, according to the manufacturer’s recommendations. The irreversible hydrocolloids were mixed vigorously by hand with distilled water in accordance with ISO 21563^18^ at room temperature for the mixing time specified by each manufacturer (ColorChange: 30 seconds; Hydrogum 5: 30 seconds; Moldeer Fast: 35 seconds) until a homogeneous mixture was obtained.

Each material was loaded into the same size 5 perforated stock tray (Tecnodent, Indaiatuba, Brazil), used throughout the study, ensuring complete coverage of the dental arch with approximately 5 mm of additional material beyond the teeth. The loaded tray was seated on the maxillary model within the respective working time for each material (ColorChange: 1 min 30 s; Hydrogum 5: 1 min 5 s; Moldeer Fast: 1 min 20 s). Each impression remained in contact with the model for the manufacturer-recommended in-mouth setting time (ColorChange: 60 s; Hydrogum 5: 45 s; Moldeer Fast: 40 s). After completion of the total setting time (ColorChange: 2 min 30 s; Hydrogum 5: 1 min 50 s; Moldeer Fast: 2 min), the impressions were carefully removed and rinsed under running water for 30 s.^19^ Excess surface water was gently blotted with paper towels.

A size 5 stainless-steel stock tray was selected according to the dimensions of the reference model to ensure an appropriate fit. The peripheral stops of the model acted as a physical seating limit, preventing overinsertion and promoting a clinically uniform alginate thickness throughout the impression. For every impression, the tray handle was aligned with the model midline to standardize positioning and improve reproducibility. Although a stock tray was used, the tray selection and seating protocol followed the same principles commonly applied to custom trays. Because of the close adaptation between the tray and the model, neither a custom tray nor spacers were required for this in vitro protocol.

### Dimensional stability evaluation

Each irreversible hydrocolloid impression was scanned once by a second experienced operator (IF) using a Medit i700 intraoral scanner (Medit Corp., Seoul, Republic of Korea) operating in regular resolution mode. The scanner was calibrated before image acquisition. Each impression was then individually stored in a sealed ziplock bag containing a moist sponge without direct contact with the impression to maintain 100% relative humidity throughout the experimental period. Blinding was not feasible because of the inherent color differences among the tested materials.

The standardized maxillary model (N51, P-OCLUSAL Produtos Odontológicos, São Paulo, Brazil), whose dimensions were known, was scanned to serve as the control (Figure 1A). Measurement landmarks were selected based on previous studies,^20,21^ and were intended to evaluate linear distances across the entire arch using clinically relevant dental landmarks: the cusp tips of the right and left canines and distal buccal cusps of the right and left first molars. These readily identifiable landmarks formed a quadrilateral in which adjacent measurement lines shared common vertices, allowing verification of landmark positioning during repeated measurements and thereby improving measurement accuracy.

**Figure 1:**
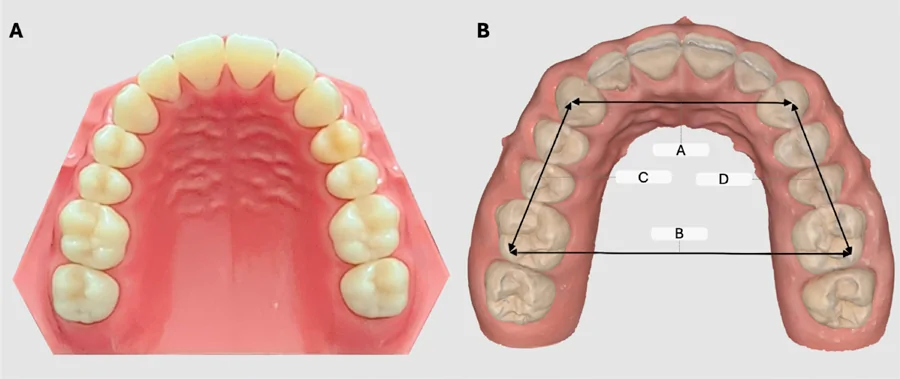
(A) Standardized maxillary model used for all irreversible hydrocolloid impressions. (B) Three-dimensional digital image of the model showing the reference landmarks and linear measurements evaluated in this study (labeled A, B, C, and D).

The measured variables were: a) intercanine distance, b) intermolar distance, c) right canine-to-molar distance, and d) left canine-to-molar distance (Figure 1B). The blinded operator (MCS) identified the measurement landmarks using the software’s point-to-point distance tool. All linear measurements were recorded in millimeters (mm) to two decimal places, consistent with the 0.01-mm measurement resolution provided by the Medit Link software.

The standardized maxillary model was scanned ten times using the same protocol to establish the reference (ground truth) dataset for measurements A–D and to determine the corresponding mean and standard deviation. Each irreversible hydrocolloid impression was scanned once at each pre-established time point: immediately after separating the tray from the model (0 h), and after 24 h, 48 h, 72 h, 96 h, and 120 h of storage.

To standardize the orientation of all scanned models, image alignment was performed using the software’s grid settings. Each STL file was oriented in the Medit Link software using a standardized protocol. The dental midline was aligned with the central vertical grid line, the occlusal surfaces were oriented perpendicular to the viewing direction to provide full visualization of the occlusal anatomy, and the central horizontal grid line was then aligned with the canine cusp tips to ensure consistent positioning of the occlusal plane across all images. A fixed zoom level was used so that the transverse dimension displayed on the screen corresponded to the transverse width of the original scanned model (approximately 7 cm), using the vestibular depth as the scaling reference.

All three-dimensional images were coded and color-masked by the researcher who performed the scanning (IF) before being exported as STL files to the blinded examiner (MCS), thereby preventing identification of the tested materials based on their original colors. Figure 2 illustrates the original negative scan and the color-masked digitally inverted positive image. All measurements were performed by the same trained, blinded examiner (MCS) using Medit Link software (version 3.3.3, Medit Corp., Seoul, Republic of Korea) (Figure 3). To assess measurement reliability and accuracy, all measurements were repeated one week later by the same examiner (MCS), yielding an intraclass correlation coefficient greater than 0.95. The recorded data were subsequently reviewed for consistency and accuracy.

**Figure 2:**
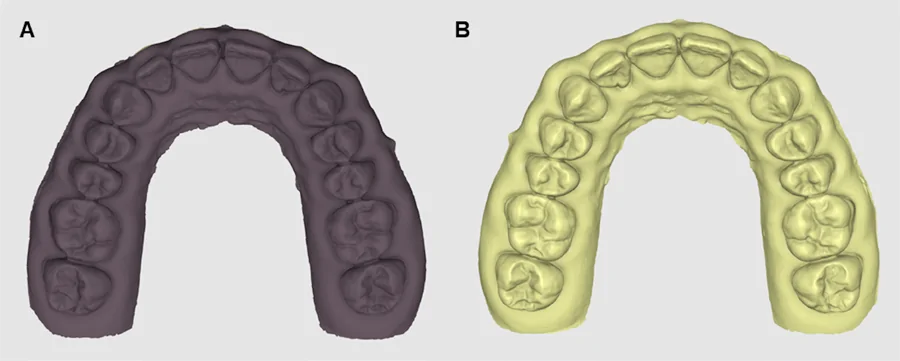
(A) Original appearance of the scanned impressions (negative). (B) Color-masked digitally inverted (positive) image.

**Figure 3:**
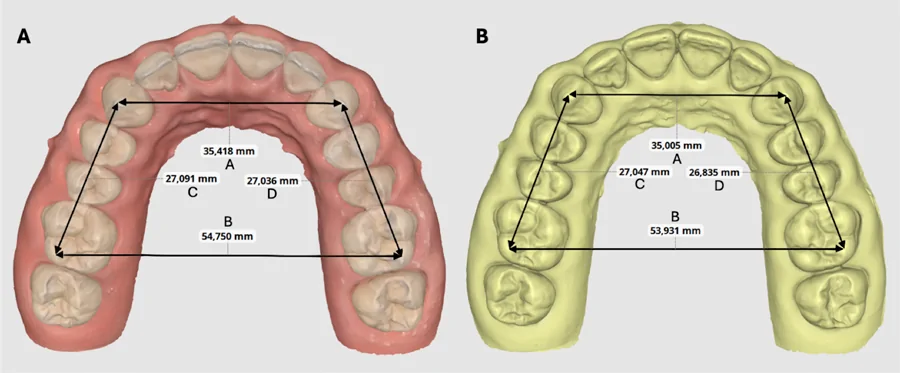
(A) Linear measurements obtained from the scanned image of the reference model in its original color. (B) Linear measurements obtained from a color-masked scanned image 24 hours after impression making.

All dimensional variations were calculated as the difference (mm) between the measurements obtained from each impression at a given time point and the corresponding measurement of the reference model:

Dimensional Variation = Measurement_impression_ – Measurement_reference_

Where:

- Negative values (−) indicate contraction (the impression is smaller than the reference model);
- Positive values (+) indicate expansion (the impression is larger than the reference model);
- Value of zero (0) indicates perfect dimensional accuracy.

### Statistical analysis

All measurements were recorded in millimeters (mm) in a Microsoft Excel spreadsheet by one researcher (MCS) and analyzed using Jamovi software (version 2.3) by a second blind researcher (LMAC). Normality was assessed using the Shapiro–Wilk test. After confirming normality, the data were analyzed using repeated-measures ANOVA with Greenhouse-Geisser correction to evaluate the effects of material and time, considering the values obtained at each time point. Repeated-measures ANOVA with Greenhouse-Geisser correction (α = 0.05) was performed for each evaluated region (A, B, C, and D) at different time points (0 h, 24 h, 48 h, 72 h, 96 h, and 120 h) to assess dimensional stability across time.

The acceptability threshold was defined according to ISO 21563:2013, which specifies that irreversible hydrocolloid impressions must maintain at least 95% dimensional accuracy relative to the original dimensions. For the measurements evaluated in this study, this criterion corresponded to maximum allowable deviations of ±1.76 mm for intercanine distance (A), ±2.74 mm for intermolar distance (B), ±1.36 mm for the right canine-to-molar distance (C), and ±1.34 mm for the left canine-to-molar distance (D).

In addition to the primary analysis of absolute deviations from the reference model, a relative-change metric was calculated for each impression and time point, defined as Δ*(t)* = *M(t)* − *M*(0 h) (mm) for each measurement (A–D). For each material and time point, relative changes were summarized as the mean ± standard deviation of the ten impressions. Net change at the end of the observation period (Δ_120 h = *M*(120 h) − *M*(0 h)) and the maximum absolute drift (peak |Δ|) were calculated for each material and measurement. This secondary analysis was intended to characterize time-dependent drift patterns while reducing dependence on the absolute reference model; however, statistical inference regarding time effects remained based on the primary repeated-measures analysis.

Box-and-whisker plots were generated for each measurement (A–D) at each of the six evaluation time points (0 h, 24 h, 48 h, 72 h, 96 h, and 120 h), with the three irreversible hydrocolloids displayed side by side at each time point. Boxes represent the interquartile range (IQR), whiskers extend to 1.5×IQR, the horizontal line indicates the median, and the dot represents the mean. These plots complement the tabulated data and facilitate visualization of the repeated-measures results.

## Results

The mean measures and the mean dimensional variations for each group at each evaluation time point are presented in Tables 2 and 3. Negative values indicate contraction, whereas positive values indicate expansion relative to the reference model.

At 0 h (immediate scanning), all materials exhibited mean values close to those of the reference model, confirming high initial dimensional accuracy. Small dimensional variations were observed over time for all four measurements (A–D).

For measurement A (intercanine distance), all irreversible hydrocolloids exhibited slight contraction throughout the evaluation period. Moldeer Fast, Hydrogum 5, and ColorChange showed only minor reductions and maintained similar values after 120 h. For measurement B (intermolar distance), no significant dimensional differences were observed among Moldeer Fast, Hydrogum 5, and ColorChange. Likewise, measurements C and D (right and left canine-to-molar distances, respectively) exhibited only minimal dimensional variation throughout the study.

Statistical analysis (Table 3) confirmed that no significant differences were observed among the materials at any evaluated time point (*p* > 0.05), indicating that all three extended-pour, fast-setting irreversible hydrocolloids maintained dimensional stability for up to 120 h. The mean reference-model values (± standard deviation) were 35.26 ± 0.10 mm for measurement A, 54.83 ± 0.19 mm for measurement B, 27.26 ± 0.29 mm for measurement C, and 26.82 ± 0.09 mm for measurement D.

**Table 2:** Mean linear measurements (mean ± standard deviation, mm) at each evaluation time point.

Measurement	Material	0 h	24 h	48 h	72 h	96 h	120 h
A	ColorChange	35.05 ± 0.22	34.98 ± 0.14	35.03 ± 0.15	35.05 ± 0.12	35.05 ± 0.09	35.11 ± 0.13
	Hydrogum 5	34.99 ± 0.32	34.94 ± 0.21	35.00 ± 0.13	34.93 ± 0.12	34.99 ± 0.11	34.88 ± 0.18
	Moldeer fast	35.02 ± 0.17	35.02 ± 0.16	34.95 ± 0.08	34.96 ± 0.11	34.92 ± 0.19	34.95 ± 0.15
B	ColorChange	54.36 ± 0.30	54.26 ± 0.21	54.28 ± 0.12	54.40 ± 0.17	54.40 ± 0.14	54.46 ± 0.10
	Hydrogum 5	54.44 ± 0.12	54.51 ± 0.16	54.49 ± 0.36	54.42 ± 0.27	54.49 ± 0.15	54.39 ± 0.15
	Moldeer Fast	54.24 ± 0.17	54.17 ± 0.20	54.36 ± 0.15	54.30 ± 0.18	54.39 ± 0.14	54.30 ± 0.21
C	ColorChange	27.01 ± 0.17	27.13 ± 0.11	27.17 ± 0.12	27.17 ± 0.08	27.09 ± 0.12	27.07 ± 0.16
	Hydrogum 5	27.14 ± 0.23	27.22 ± 0.17	27.18 ± 0.16	27.15 ± 0.12	27.21 ± 0.15	27.26 ± 0.09
	Moldeer Fast	27.02 ± 0.09	27.08 ± 0.26	27.05 ± 0.11	27.13 ± 0.11	27.14 ± 0.10	27.11 ± 0.16
D	ColorChange	26.79 ± 0.17	26.94 ± 0.13	26.96 ± 0.12	26.94 ± 0.20	26.90 ± 0.14	26.85 ± 0.15
	Hydrogum 5	26.78 ± 0.08	26.92 ± 0.14	27.01 ± 0.39	26.99 ± 0.10	26.97 ± 0.23	26.95 ± 0.31
	Moldeer Fast	26.72 ± 0.15	26.84 ± 0.15	26.84 ± 0.15	26.88 ± 0.15	26.94 ± 0.14	26.90 ± 0.16

**Table 3:** Mean linear dimensional variation relative to the reference model (mm; 95%CI) at each evaluation time point.

Measurement	Material	0 h	24 h	48 h	72 h	96 h	120 h
A	Hydrogum 5	-0.27 (0.1 to 0.4) A	-0.32 (0.2 to 0.5) A	-0.26 (0.2 to 0.4) A	-0.33 (0.2 to 0.4) A	-0.27 (0.2 to 0.4) A	-0.38 (0.3 to 0.5) A
	Moldeer fast	-0.24 (0.1 to 0.3) A	-0.24 (0.1 to 0.3) A	-0.31 (0.2 to 0.4) A	-0.24 (0.1 to 0.3) A	-0.34 (0.2 to 0.4) A	-0.31 (0.2 to 0.4) A
	Colorchange	-0.21 (0.1 to 0.4) A	-0.28 (0.2 to 0.4) A	-0.23 (0.1 to 0.3) A	-0.21 (0.1 to 0.3) A	-0.19 (0.0 to 0.3) A	-0.15 (0.1 to 0.2) A
B	Hydrogum 5	-0.39 (0.3 to 0.5) A	-0.32 (0.2 to 0.4) A	-0.34 (0.1 to 0.5) A	-0.41 (0.3 to 0.6) A	-0.34 (0.2 to 0.4) A	-0.44 (0.3 to 0.5) A
	Moldeer fast	-0.59 (0.4 to 0.8) A	-0.66 (0.5 to 0.8) A	-0.47 (0.4 to 0.6) A	-0.53 (0.4 to 0.6) A	-0.44 (0.3 to 0.5) A	-0.53 (0.4 to 0.7) A
	ColorChange	-0.47 (0.3 to 0.7) A	-0.57 (0.4 to 0.7) A	-0.55 (0.5 to 0.6) A	-0.43 (0.3 to 0.5) A	-0.43 (0.3 to 0.5) A	-0.37 (0.3 to 0.5) A
C	Hydrogum 5	-0.12 (-0.1 to 0.4) A	-0.04 (-0.2 to 0.2) A	-0.08 (-0.1 to 0.3) A	-0.11 (-0.1 to 0.3) A	-0.05 (-0.2 to 0.3) A	0.0 (-0.2 to 0.2) A
	Moldeer fast	-0.24 (0.0 to 0.4) A	-0.18 (-0.1 to 0.4) A	-0.21 (0.0 to 0.4) A	-0.13 (-0.1 to 0.3) A	-0.12 (-0.1 to 0.3) A	-0.15 (-0.1 to 0.4) A
	Colorchange	-0.25 (0.0 to 0.5) A	-0.13 (-0.1 to 0.3) A	-0.09 (-0.1 to 0.3) A	-0.09 (-0.1 to 0.3) A	-0.17 (0.0 to 0.4) A	-0.19 (0.0 to 0.4) A
D	Hydrogum 5	-0.04 (0.0 to 0.1) A	0.1 (-0.2 to 0.0) A	0.19 (-0.4 to 0.0) A	0.17 (-0.3 to -0.1) A	0.15 (-0.3 to 0.0) A	0.13 (-0.3 to 0.1) A
	Moldeer fast	-0.1 (0.0 to 0.2) A	0.02 (-0.1 to 0.1) A	0.02 (-0.1 to 0.1) A	0.06 (-0.1 to 0.0) A	0.12 (-0.2 to 0.0) A	0.08 (-0.2 to 0.0) A
	Colorchange	-0.03 (-0.1 to 0.1) A	0.12 (-0.2 to 0.0) A	0.14 (-0.2 to -0.1) A	0.12 (-0.3 to 0.0) A	0.08 (-0.2 to 0.0) A	0.03 (-0.1 to 0.1) A

Repeated-measures ANOVA followed by the Greenhouse-Geisser correction. Identical uppercase letters indicate no statistically significant differences among time points (p > 0.05).

The transverse distances between the canines (A) and first molars (B), as well as the longitudinal right canine-to-molar distance (C), exhibited fluctuations between contraction and expansion, with no statistically significant differences observed throughout the 120-h evaluation period (p > 0.05). Although dimensional changes fluctuated, most values were negative, indicating slight material contraction over time, without statistically significant differences at any evaluated time point. For the left canine-to-molar distance (D), all materials exhibited slight contraction at 0 h, which was less pronounced than that observed for the other measurements. Over time, fluctuations between expansion and contraction were observed, but predominantly positive values were recorded, suggesting minimal expansion in this dimension. However, no statistically significant differences were found at any time point (p > 0.05).

Comparisons among the materials at the initial (0 h; Table 4) and final (120 h; Table 5) evaluation time points demonstrated similar overall dimensional behavior. An exception was observed for measurement A at 120 h, in which Hydrogum 5 exhibited greater dimensional contraction relative to the reference model (control), while Moldeer Fast showed intermediate values comparable to those of the reference model, ColorChange, and Hydrogum 5.

**Table 4:** Mean linear dimensional variation (mean ± standard deviation, mm) at 0 h.

Variable	A	B	C	D
ColorChange	-0.21 ± 0.22 A	-0.46 ± 0.29 A	-0.24 ± 0.26 A	-0.03 ± 0.17 A
Hydrogum 5	-0.27 ± 0.31 A	-0.38 ± 0.12 A	-0.12 ± 0.36 A	-0.04 ± 0.13 A
Moldeer fast	-0.24 ± 0.17 A	-0.59 ± 0.17 A	-0.24 ± 0.29 A	-0.10 ± 0.20 A

One-way ANOVA followed by Tukey’s test. Identical uppercase letters indicate no statistically significant differences among time points (p > 0.05).

**Table 5:** Mean linear dimensional variation (mean ± standard deviation, mm) at 120 h.

Variable	A	B	C	D
ColorChange	-0.15 ± 0.13 A	-0.36 ± 0.10 A	-0.18 ± 0.28 A	0.03 ± 0.17 A
Hydrogum 5	-0.38 ± 0.18 B	-0.43 ± 0.14 A	0.00 ± 0.33 A	0.13 ± 0.30 A
Moldeer fast	-0.31 ± 0.14 AB	-0.53 ± 0.20 A	-0.15 ± 0.41 A	0.08 ± 0.20 A

One-way ANOVA followed by Tukey’s test. Distinct uppercase letters indicate statistically significant differences among time points (p ≤ 0.05).

When expressed as relative change from 0 h (Δ), the magnitudes of the drifts remained small across all materials and measurements, ranging from −0.11 to +0.23 mm over the 120-h period, with no consistent monotonic trend. This finding is consistent with the absence of significant time effects in the repeated-measures analysis.

The box-and-whisker plots for A–D (Figures 4 and 5) demonstrated tightly clustered, nonmonotonic distributions across all six evaluation time points for the three irreversible hydrocolloids. These visual findings are consistent with the absence of statistically significant time effects observed in the repeated-measures analysis. Minor oscillations over time remained well within the variability represented by the mean ± standard deviation and 95% confidence intervals reported in the tables.

## Discussion

In dentistry, new materials are continually being developed to preserve or improve established properties while introducing additional clinical advantages. Recently, Moldeer Fast was introduced as a mint-flavored color-changing, fast-setting, extended-pour irreversible hydrocolloid designed for compatibility with digital workflows. The present study evaluated its dimensional stability compared with two widely used and well-established materials: ColorChange and Hydrogum 5. No statistically significant differences were observed among the tested materials, thereby supporting the null hypothesis.

Moldeer Fast demonstrated minimal dimensional variation comparable to that of ColorChange and Hydrogum 5. These findings support the generation of accurate digital models through direct scanning of irreversible hydrocolloid impressions for up to five days after impression preparation. This extended dimensional stability eliminates the need for immediate cast fabrication and facilitates integration into digital workflows. While the present results support flexibility in scheduling digital scanning, extrapolation of these findings to delayed gypsum pouring should be interpreted cautiously, since impression–gypsum interactions during pouring and cast separation were not evaluated.

To optimize the experimental design, a standard maxillary typodont occlusal model with known dimensions was used to better reproduce clinical conditions while maintaining methodological control. Dimensional changes were evaluated by serial direct scanning of the impressions rather than by scanning gypsum casts. This approach provides a more accurate representation of the dimensional behavior of the impression materials while minimizing the influence of additional materials^22^. Scanning gypsum casts may introduce additional variability resulting from interactions between the impression material and the dental stone,^22^ thereby affecting dimensional accuracy. Furthermore, the increasing availability of intraoral scanners in clinical practice makes this approach progressively more practical. In addition to improving treatment planning, digital models reduce physical storage requirements and facilitate communication between clinicians and dental laboratories.

**Figure 4:**
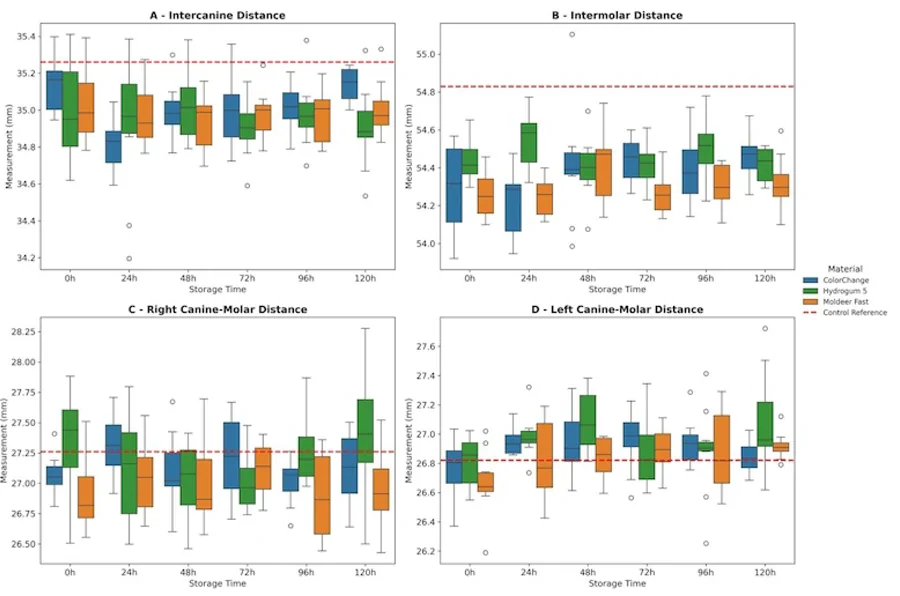
Linear measurements (mm) over time (A–D).

Boxes = interquartile range (IQR); whiskers = 1.5 × IQR; center line = median; dot = mean.

Interpretation of the dimensional stability findings was based on ISO 21563:2013,^18^ which specifies that irreversible hydrocolloid impressions must maintain at least 95% dimensional accuracy. Within this framework, all tested materials demonstrated clinically acceptable dimensional stability for direct digital scanning throughout the evaluation period. Previous studies have likewise reported comparable dimensional stability for extended-pour irreversible hydrocolloids,^2,5-8,11-17^ supporting their clinical reliability over a five-day period. The slight dimensional contraction observed for all materials is consistent with the expected effects of water evaporation^4^ and the limited capacity of the gel matrix to retain water after mixing.^23^ A noteworthy finding for clinical practice is that small differences in the water-to-powder ratios recommended by the manufacturers, together with storage under controlled humidity, likely contributed to the similar performance among the materials. Moreover, the use of a trained operator and maintenance of the impressions within the trays throughout the evaluation period probably helped preserve dimensional stability. To minimize tearing and deformation, impressions should remain in their trays until use or disposal.

Clinical practice is undergoing a transition toward digital workflows; however, irreversible hydrocolloids remain relevant in routine dental practice. The present findings support the use of irreversible hydrocolloids digitized using the present protocol for study models, preliminary records, orthodontic assessments, antagonist casts, bleaching trays, interocclusal devices (e.g., myorelaxant splints), removable appliances, individual impression trays, surgical guides, and removable partial denture workflows—applications in which small linear deviations are generally acceptable for diagnosis, treatment planning, or device fabrication. A further aspect that warrants consideration is the use of an intraoral scanner for full-arch acquisitions combined with manual placement of measurement landmarks, which can increase measurement variability and obscure subtle contraction or expansion attributable to the impression materials. Previous studies have shown that, in full-arch acquisitions, datasets obtained with intraoral scanners may exhibit lower trueness and/or precision than those acquired with dedicated extraoral or model scanners, thereby contributing to variability in linear measurements.^24^ To minimize these sources of uncertainty, several standardization and quality-control procedures were implemented: a) standardized model orientation and visualization using the software grid; b) consistent placement of predefined, clinically relevant landmarks using the point-to-point measurement tool; c) all measurements performed by a trained, blinded examiner, with repeat assessments conducted after one week and a high intraclass correlation coefficient confirming repeatability; d) controlled storage conditions to minimize handling-related variability; and e) a repeated-measures ANOVA design, in which each specimen served as its own control, thereby minimizing the influence of systematic measurement error on comparisons among materials and time points.

**Figure 5:**
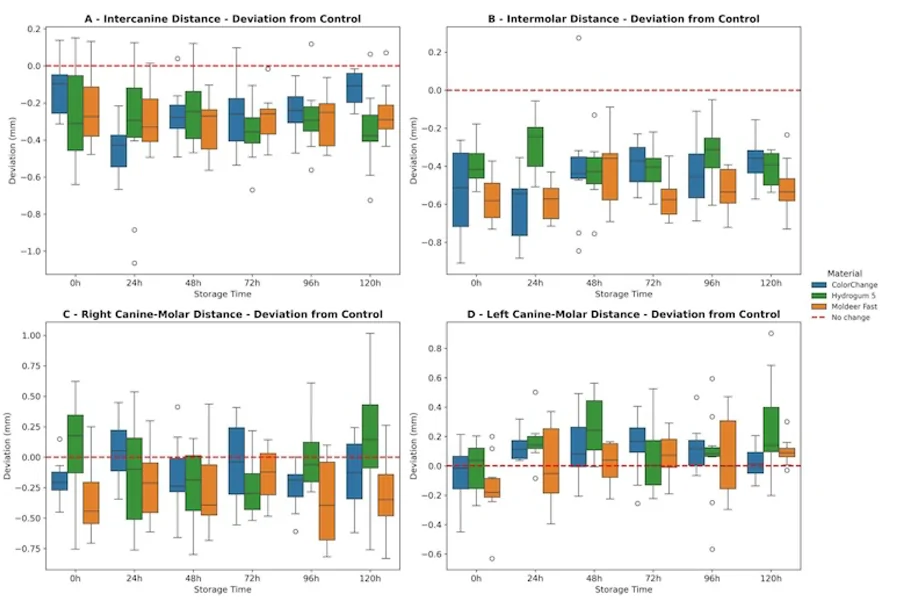
Deviation from control (mm) over time (A–D).

Boxes = interquartile range (IQR); whiskers = 1.5 × IQR; center line = median; dot = mean.

As a complementary robustness assessment, the relative-change analysis demonstrated small, non-monotonic drifts over time, corroborating the absence of significant time effects and supporting the interpretation that these minor oscillations fall within the expected measurement variability associated with full-arch intraoral scanning under the present protocol. Despite these measures, some scanner- and operator-dependent variability cannot be excluded. The selection of an intraoral scanner was intentional, since the study sought to reproduce a common clinical workflow in which the scanner already available in the dental office is used to digitize impressions. Future studies should investigate the use of dedicated impression or model scanners with higher full-arch accuracy, as well as automated or template-guided landmark identification methods to further reduce measurement error.

Although this study provides valuable information regarding the dimensional behavior of extended-pour, fast-setting irreversible hydrocolloids, certain limitations should be acknowledged. As an in vitro study, it did not reproduce intraoral conditions such as temperature and humidity variations during setting. In addition, the evaluation was limited to direct digital scanning of impressions and did not include comparisons with gypsum casts or assessments involving multiple operators. Future investigations should evaluate the effects of different storage conditions (e.g., temperature and relative humidity) on dimensional stability, compare immediate and delayed scanning protocols, evaluate the influence of interoperator variability, and include clinical studies evaluating factors such as saliva contamination, intraoral temperature, user satisfaction, and handling under clinical conditions.

## Conclusions

Moldeer Fast exhibited dimensional stability comparable to that of the extended-pour, fast-setting irreversible hydrocolloids ColorChange and Hydrogum 5 under the conditions of the present in vitro scanning protocol. No statistically significant time effects were observed over the 120-h evaluation period, and the dimensional variations remained small across all measurements. These findings support its use as a high-performance impression material capable of maintaining dimensional stability for up to five days. Furthermore, the results validate its compatibility with digital scanning, reinforcing its applicability within contemporary digital workflows while maintaining clinically acceptable dimensional accuracy over time.

## Data Availability

The authors declare that all data generated or analyzed during this study are included in this published article.
